# Energy Donor Effect on the Sensing Performance for a Series of FRET-Based Two-Photon Fluorescent Hg^2+^ Probes

**DOI:** 10.3390/ma10020108

**Published:** 2017-01-25

**Authors:** Yujin Zhang, Wei Hu

**Affiliations:** 1 School of Science, Qilu University of Technology, Jinan 250353, China; zhangyujin312@163.com; 2 Hefei National Laboratory for Physical Sciences at the Microscale, iChEM (Collaborative Innovation Center of Chemistry for Energy Materials), School of Chemistry and Materials Science, University of Science and Technology of China, Hefei 230026, China

**Keywords:** two-photon fluorescent probe, two-photon absorption, two-photon microscopy

## Abstract

Nonlinear optical properties of a series of newly-synthesized molecular fluorescent probes for Hg^2+^ containing the same acceptor (rhodamine group) are analyzed by using time-dependent density functional theory in combination with analytical response theory. Special emphasis is placed on evolution of the probes’ optical properties in the absence and presence of Hg^2+^. These compounds show drastic changes in their photoabsorption and photoemission properties when they react with Hg^2+^, indicating that they are excellent candidates for ratiometric and colorimetric fluorescent chemosensors. Most importantly, the energy donor moiety is found to play a dominant role in sensing performance of these probes. Two-photon absorption cross sections of the compounds are increased with the presence of Hg^2+^, which theoretically suggests the possibility of the probes to be two-photon fluorescent Hg^2+^ sensors. Moreover, analysis of molecular orbitals is presented to explore responsive mechanism of the probes, where the fluorescence resonant energy transfer process is theoretically demonstrated. Our results elucidate the available experimental measurements. This work provides guidance for designing efficient two-photon fluorescent probes that are geared towards biological and chemical applications.

## 1. Introduction

Mercury is one of the most toxic heavy metal elements [1,2]. Studies have shown that exposure to mercury, even at very low concentrations, leads to various diseases in the digestive system, the nervous system, and especially the neurological system, which makes detection of mercury ions (Hg^2+^) an attractive research area in the biological field [3,4]. Thus, much attention has been focused on developing new methods to monitor Hg^2+^ in biological and environmental samples [5,6,7]. Among various kinds of methods, the fluorescent probe method shows important application value because of its high sensitivity, high selectivity, and low damage to samples [8]. In this context, fluorescent probes combined with fluorescence microscopy provide an efficient approach to determining the physiological activity components in real time [9,10]. As a consequence, the design of chemosensors for Hg^2+^ becomes an attractive subject in many fields.

There are several signaling mechanisms that can be employed for designing sensitive fluorescent molecular probes [11,12], such as intramolecular charge transfer (ICT) [13], excimer/exciplex formation [14], fluorescence resonance energy transfer (FRET) [15,16,17] and through-bond energy transfer (TBET). FRET is a nonradiative energy transfer process in which the excitation energy is transferred from the energy donor to the nearby energy acceptor via the long-range dipole–dipole interaction and/or short-range multipolar interaction. This strategy could provide moderate resolution of the two emission bands and has been widely applied in designing ratiometric probes for bioimaging applications [18].

Over the last decade, many experimentalists have been involved in searching for efficient fluorescent FRET-based Hg^2+^ probes that can be used in the fluorescent imaging technology [19,20]. Very recently, Liu et al. have synthesized a new naphthalimide-rhodamine fluorescent probe for Hg^2+^ (named hereafter as Pro1) based on an intramolecular FRET with an excellent selectivity over other metal ions [21]. By adopting the same energy acceptor of Pro1, Zhang et al. have developed another FRET-based probe (named hereafter as Pro2) that can selectively detect amounts of Hg^2+^ ions on the ppb scale under physiological conditions [22]. Moreover, an irreversible Hg^2+^ selective ratiometric fluorescence probe (named hereafter as Pro3), composed of a fluorescein fluorophore linked to a rhodamine B hydrazide by a thiourea spacer, was designed and synthesized by Shang et al. [23].

The experiments mentioned above have shown that the probes Pro1, Pro2 and Pro3 have high efficiency for detecting Hg^2+^. However, which one is preferable remains an open question. Moreover, although the responsive mechanism for this probe is discussed in the experiments, the first principle studies and theoretical analyses on this issue are still insufficient and not satisfying. Above all, the measurements were performed by one-photon excitation, and these probes were investigated at one-photon level. As is well known, a one-photon fluorescent probe has disadvantages with interferences from self-absorption, auto-fluorescence and photodamage [24,25,26]. If these probes can be used as two-photon fluorescent probes, one can expect that these probes would be extended for wider application. In this paper, we carry out theoretical studies on the one-photon absorption (OPA) and two-photon absorption (TPA) as well as fluorescent properties of the probes Pro1, Pro2 and Pro3 in the absence and presence of Hg^2+^. By discussing the optical properties of the probes, their sensing performances are compared. Special attention is paid to the analysis of responsive mechanisms of the probes by illustrating molecular orbital distributions of the compounds involved in the absorptive and emissive processes. The present research would be helpful to understand the relationships between the structure and the optical properties for these FRET-based fluorescent probes. Most of all, guidelines for designing more efficient two-photon fluorescent probes are provided based on the present theoretical studies.

## 2. Theoretical Methods and Computational Details

### 2.1. Theoretical Methods

Details of the theoretical methods are referred to in [27]. Here, we just list the main formulations. The transition probability of one-photon absorption and emission can be described by the oscillator strength:

(1) $$\delta_{op}=\frac{2\omega_{f}}{3}{\sum_{\alpha }\left|\langle 0\left|\mu_{\alpha }\right|f\rangle \right|}^{2}$$

where ω_*f*_ denotes the excitation energy of the excited state *f*, μ_α_ is the electric dipole moment operator, and the summation is performed over *x*, *y*, *z* axes.

The TPA cross section that can be compared directly with the experimental value is defined as

(2) $$\sigma_{tpa}=\frac{4\pi^{2}a_{0}^{5}\alpha }{15c}\frac{\omega^{2}g\left(\omega \right)}{\Gamma_{f}}\delta_{tpa}$$

where *a*_0_, α, *c* is the Bohr radius, the fine structure constant, and the light speed, respectively. *ħ*ω is the photon energy of the incident light. *g*(ω) is the spectral line profile, which is assumed to be a δ function here, and Γ_*f*_ is level broadening of the final state, which is taken as a typical value of 0.1 eV [28]. δ_*tpa*_ is the orientational averaging value of the TPA probability [27].

### 2.2. Computational Details

The geometries of all the compounds are optimized at the time-dependent hybrid density functional theory (TD-DFT)/Becke’s three parametrized Lee-Yang-Parr (B3LYP) exchange functional level utilizing the Gaussian09 program (Gaussian Inc., Wallingford, CT, USA) [29]. Frequency calculations are then performed to verify the stability of optimized structures. Based on the ground state structures of the compounds, OPA and TPA properties are investigated using quadratic response theory implemented in the Dalton2013 package [30]. To study the fluorescent emission properties of the compounds, we optimized the first excited states of the compounds using the Gaussian09 program. The basis set of 6-31G(d) is chosen for all of the studies. Furthermore, the polarizable continuum model is employed to consider the solvent effect of water during the whole study.

## 3. Results and Discussion

### 3.1. Molecular Structure

**Figure 1:**
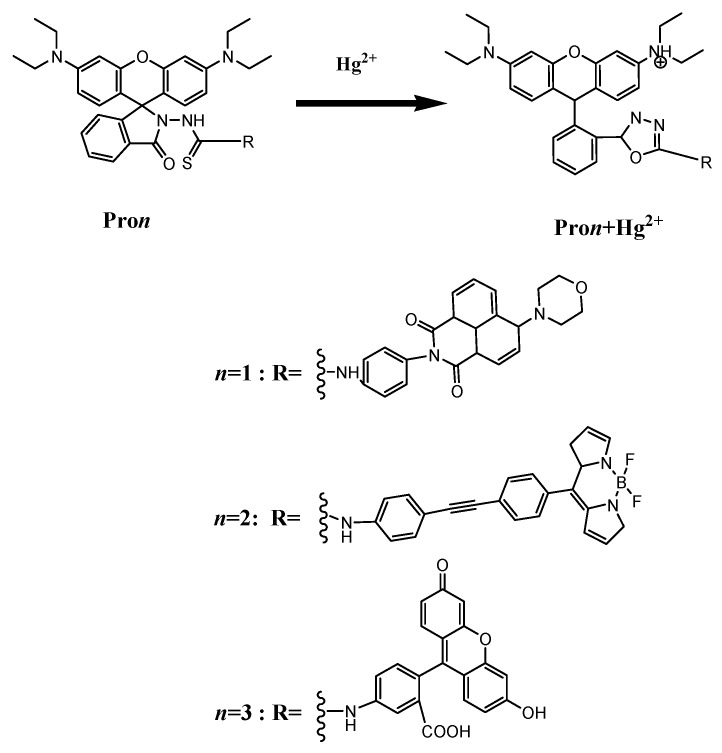
Molecular structures of Pro1, Pro1+Hg^2+^, Pro2, Pro2+Hg^2+^, Pro3 and Pro3+Hg^2+^.

**Figure 2:**
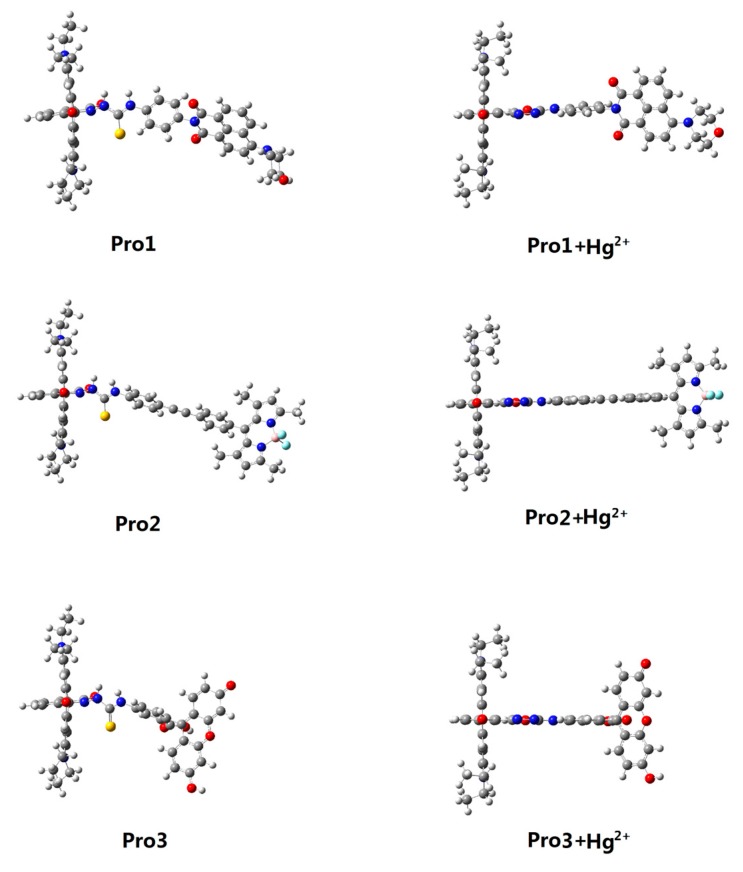
Optimized ground state geometries of Pro1, Pro1+Hg^2+^, Pro2, Pro2+Hg^2+^, Pro3 and Pro3+Hg^2+^ in H_2_O.

Structural formulas of the studied compounds are depicted in Figure 1. Compounds of Pro1, Pro2 and Pro3 possess the same energy acceptor (rhodamine moiety), which can be chemically modified by Hg^2+^. With the presence of Hg^2+^, the energy acceptor (rhodamine moiety) exists in an open-ring form as shown in Pro1+Hg^2+^, Pro2+Hg^2+^ and Pro3+Hg^2+^. On the other hand, Pro1, Pro2 and Pro3 contains different energy donors, namely, naphthalimide derivative for Pro1, boron-dipyrromethene (BODIPY) derivative for Pro2, and fluorescein derivative for Pro3, respectively. The different energy donors would affect the OPA and TPA properties of the compounds [31,32,33,34]. The optimized ground state geometries of the compounds in H_2_O are shown in Figure 2. It can be seen that there is a large tortuosity between the energy acceptor and energy donor of the probes. Thus, one can expect from the probes’ non-coplaner features that the compounds would not behave as a single conjugated dye and the rapid energy transfer process from the donor to the acceptor is easy to occur.

### 3.2. One-Photon Absorption

In the experiment, Pro1, Pro2 and Pro3 show featured absorption peaks at 405 nm, 501 nm and 490 nm, respectively. With the addition of Hg^2+^, new strong absorption peaks with wavelengths of significant redshifts were observed [21,22,23]. In the present work, we firstly study the optical absorption properties of the compounds systematically. To be specific, OPA properties of the compounds in H_2_O, including the excitation energies, the corresponding wavelengths, oscillator strengths and transition natures are calculated, and the results together with the experimental measurements are listed in Table 1.

It demonstrates that the maximum absorption peak of Pro1 is located at 434 nm with the oscillator strength of 0.35, which is attributed to the transition from the HOMO-2 to the LUMO (here, HOMO and LUMO represent the highest occupied molecular orbital and the lowest unoccupied molecular orbital, respectively). When Hg^2+^ is present, two main electronic transitions are allowed for Pro1+Hg^2+^, locating at 489 nm and 434 nm with respective oscillator strength of 0.88 and 0.37. Here, the two peaks appeared in the OPA spectrum of Pro1+Hg^2+^ origin from the HOMO-1 to LUMO and HOMO to LUMO+1 transitions, respectively. It is noted that the transition from HOMO to LUMO+1, resulting in the adsorption peak at 434 nm is exactly the same as the one of Pro1, corresponding to the transition inside the energy donor. On the other hand, the peak at 489 nm results from the transition inside the energy acceptor. For Pro2 and Pro3, absorption peaks of the compounds are located at 442 and 436 nm, respectively. After reacting with Hg^2+^, both Pro2+Hg^2+^ and Pro3+Hg^2+^ show two absorption bands, i.e., 491 (440) nm with the oscillator strength of 0.96 (0.58) for Pro2+Hg^2+^, and 495 (426) nm with the oscillator strength of 0.73 (0.35) for Pro3+Hg^2+^. Similar to the case in Pro1, both Pro2+Hg^2+^ and Pro3+Hg^2+^ show new adsorption peaks comparing to Pro2 and Pro3.

The newly appeared peaks of Pro1+Hg^2+^, Pro2+Hg^2+^ and Pro3+Hg^2+^ look like redshifts, which is well consistent with the corresponding experimental results in trends [21,22,23]. It should be noted that, although the theoretical results for different molecules show the same trends as the experimental measurements, there exists quantitative discrepancy between the calculated and experimental values. This discrepancy may mainly result from a few factors, such as the vibrational contribution, the interaction between laser and molecules, etc. It is clear that the probes Pro1, Pro2 and Pro3 display obviously different absorption performances both in the absence and in the presence of Hg^2+^, which can be attributed to the different energy donors of the probes [31].

### 3.3. Two-Photon Absorption

As we have mentioned above, the experiments are performed upon one-photon excitation. It is valuable to explore whether the probes can be used as two-photon fluorescent probes. As is known, an excellent two-photon fluorescent probe should show enhancement in the TPA cross section when it detects the analyte. Thus, the calculated results of TPA including the excitation energies, the corresponding two-photon wavelengths, and TPA cross sections of the lowest five excited states for all the compounds in H_2_O solvent are collected in Table 2. It demonstrates that the largest TPA cross sections of Pro1 and Pro1+Hg^2+^ are 534.72 GM and 2502.89 GM, located at 868 nm and 802 nm, respectively. Moreover, the maximum TPA cross section values of the probes Pro2 and Pro3 are 108.08 GM and 89.22 GM, located at 899 nm and 873 nm, respectively. With the addition of Hg^2+^, the maximum TPA cross sections are, respectively, 225.73 GM and 142.91 GM for Pro2+Hg^2+^ and Pro3+Hg^2+^. It is seen that the TPA cross section values of the compounds are enlarged to different extents when the probes react with Hg^2+^, especially in Pro1. In addition, the maximum TPA cross sections follow the order of Pro1(+Hg^2+^) > Pro2(+Hg^2+^) > Pro3(+Hg^2+^).

Note that the TPA cross section of Pro1 is much larger than that of Pro2 and Pro3 both in the absence and presence of Hg^2+^. Due to the compounds possessing the same energy acceptor but different energy donors, it can be concluded that the energy donor plays a dominant role in TPA performance of the probes [32,33]. As a consequence, the naphthalimide derivative is proved to be a preferable candidate as the TPA fluorophore in comparison with the BODIPY derivatives and the fluorescein derivatives. One can expect from the TPA results above that these probes exhibit TPA enhancement when they react with Hg^2+^. Furthermore, Pro1 has the largest TPA cross section, indicating that it can be used as a preferable two-photon fluorescent probe.

### 3.4. Fluorescent Emission

Great changes in the fluorescent spectra of the probes were experimentally observed when Hg^2+^ was added [21,22,23]. With the addition of Hg^2+^ into the probes-containing solution, the donor’s characteristic emission peak gradually disappeared, while the characteristic emission peak of rhodamine grew, indicating an efficient energy transfer process upon the reaction. Emission properties of Pro1, Pro1+Hg^2+^, Pro2, Pro2+Hg^2+^, Pro3 and Pro3+Hg^2+^, including the emission energies, the corresponding emission wavelengths, oscillator strengths, and transition natures in H_2_O solution are calculated based on the optimized first excited state geometries of the compounds.

As is clearly seen in Table 3, the calculated emission peak for Pro1 is located at 478 nm with the oscillator strength of 0.37, which can be attributed to the LUMO to HOMO-2 transition of the optimized first excited state. In comparison with Pro1, the emission wavelength of Pro1+Hg^2+^ is redshifted to 535 nm with a stronger oscillator strength of 1.14. For Pro2, upon the addition of Hg^2+^, the emission wavelength is redshifted from 489 nm to 538 nm, which appears to be in reasonable agreement with the experimental data [22]. Except for the redshift feature, the fluorescent intensity is increased from 0.52 to 1.23 when Pro2 reacts with Hg^2+^. Similarly, with the titration of Hg^2+^ into Pro3, the emission position is significantly redshifted and the emission intensity is greatly increased with respect to the free Pro3. These calculated results show that emission peak positions for the three compounds are obviously redshifted with the presence of Hg^2+^, which is in good agreement with the experimental observations [21,22,23]. Thus, the simulations prove that Pro1, Pro2 and Pro3 can be used as promising ratiometric fluorescent chemosensors. In particular, Pro1 exhibits the most remarkable changes both on the fluorescent spectrum shift and on the fluorescent intensity enhancement compared with Pro2 and Pro3, suggesting it to be the superior fluorescent probe.

### 3.5. Responsive Mechanism

The orbital distribution diagram is shown to be a useful tool for analyzing the responsive process of a molecule to the exciting light [35]. Thus, the distributions of molecular orbitals involved in the absorptive and emissive processes of the probes both in the absence and presence of Hg^2+^ are displayed to explore the responsive mechanism of the probes.

**Figure 3:**
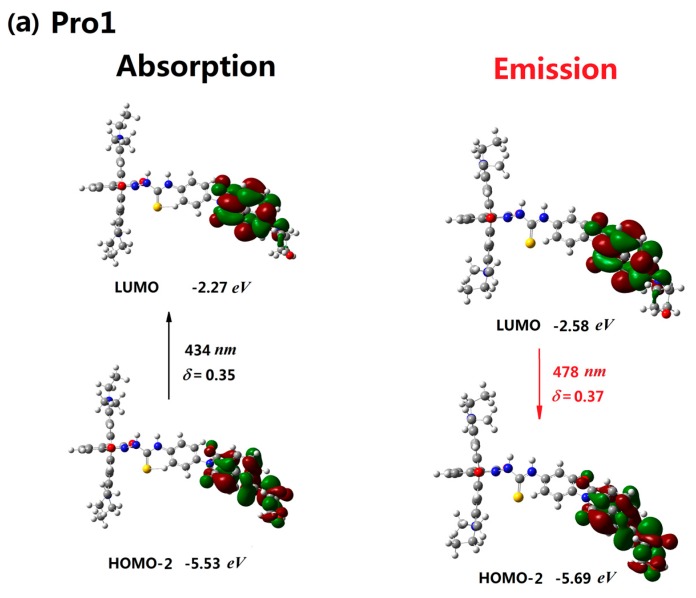

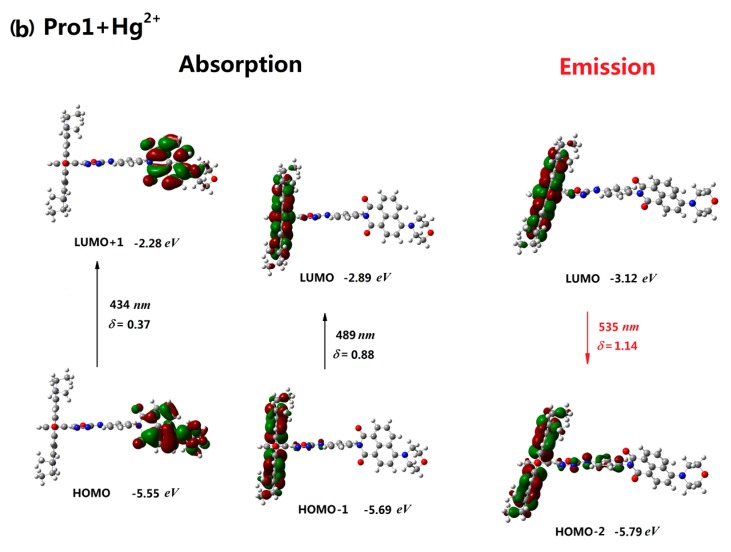
The absorption and emission of (**a**) Pro1 and (**b**) Pro1+Hg^2+^ in H_2_O.

The absorptive and emissive processes of Pro1 are presented in Figure 3a. When excited by the incident light, the mainly allowed electronic transition of Pro1, which originates from HOMO-2 to LUMO transition (434 nm, see Table 1), is mainly distributed on the energy donor moiety. Thus, the process of the absorption of Pro1 is mainly localized on the energy donor moiety (see Figure 3a). After reaching the excited state, the molecular configuration will change rapidly through the conformation transformation process. Then, the subsequent internal conversion and vibrational relaxation leads Pro1 to be located at the lowest vibrational level on the first excited state. It is noted that this process is very fast and non-radiative. Finally, the molecule will decay back to the ground state with the photon emission according to the Kasha’s rule. The ab initio calculated results in Table 3 show that the emission of Pro1 is attributed to the LUMO to HOMO-2 transition of the first excited state (478 nm). Due to the fact that both the LUMO and HOMO-2 of the first excited state of Pro1 are distributed on the energy donor moiety, the emission process of Pro1 is also distributed on the energy donor part (see Figure 3a). Comparing the adsorption and emission of Pro1, we can find that these two processes are definitely opposite. However, due to the fact that the geometries of the ground and first excited state vary a little, the energy of the emitted photon is a little smaller than the adsorbed one.

In the presence of Hg^2+^, the responsive process of the molecule to the light changes a lot. Generally, there are two main allowed electronic transitions for Pro1+Hg^2+^ upon excitation, i.e., from the HOMO to the LUMO+1 (434 nm) and from the HOMO-1 to the LUMO (489 nm). Different from Pro1, these two transitions are localized on the energy donor and energy acceptor moiety, respectively (see Figure 3b). Obviously, the later one is newly appeared and is caused by Hg^2+^. More importantly, this transition shows a larger transition dipole moment and a discrepant wavenumber compared to the one already existing in Pro1. This is the underlying reason why Pro1 can be used as a probe. By now, we can conclude several principles of designing an efficient probe: (1) easily reacts with the detect target; (2) the geometric and electronic structure of the probe should be modified by the target to emerge new transitions; (3) new transitions should exhibit high oscillator strengths and discrepant wavelengths. 

With respect to the emission of Pro1+Hg^2+^, it can be seen from the distributions of the molecular orbitals that the emissive process of Pro1+Hg^2+^ resulting from the LUMO to HOMO-2 transition is localized on the energy acceptor unit (535 nm, see Figure 3b). Based on the discussion above, we can conclude that if Pro1+Hg^2+^ is exposed in the light with the wavelength of 434 nm, it can be excited to a higher excited state, corresponding to an electronic transition on the energy donor moiety. However, the emission of the molecule originating from the decay from the first excited state to the ground state of the molecule is located at the energy acceptor unit with the fluorescence wavelength of 535 nm. Thus, the locations of the adsorption and emission for Pro1+Hg^2+^ are different, meaning that a nonradiative energy transfer process occurs. This process was named as the fluorescence resonance energy transfer (FRET), which was first proposed by Förster [36].

**Figure 4:**
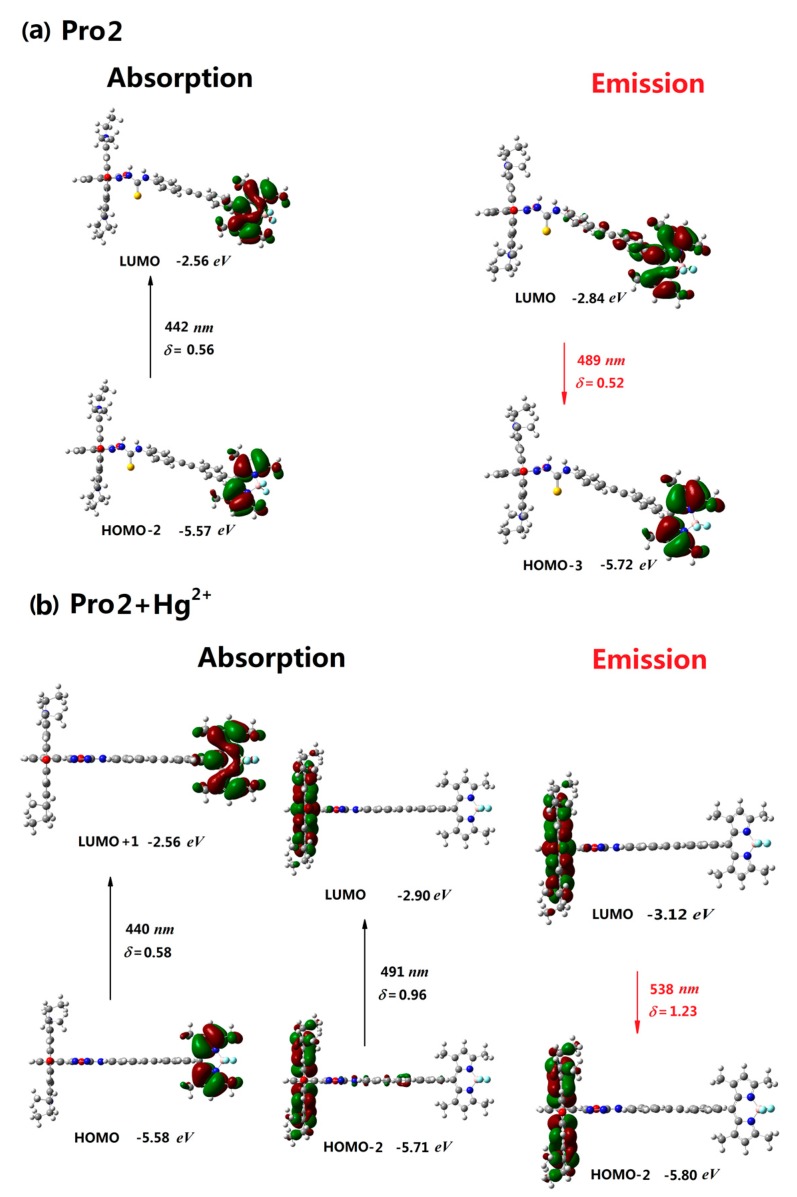
The absorption and emission of (**a**) Pro2 and (**b**) Pro2+Hg^2+^ in H_2_O.

**Figure 5:**
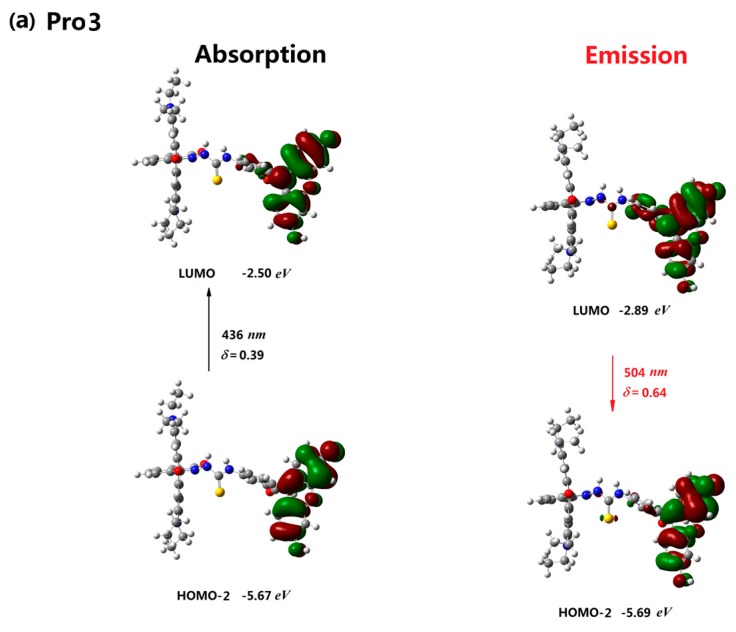

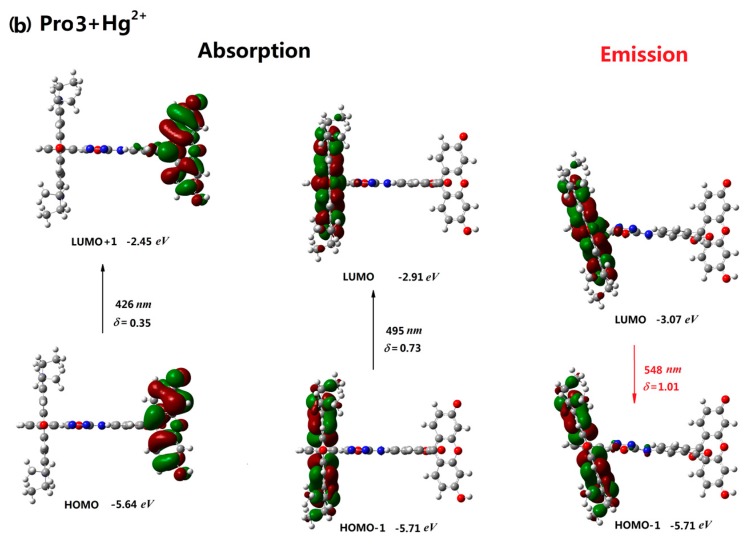
The absorption and emission of (**a**) Pro3 and (**b**) Pro3+Hg^2+^ in H_2_O.

The molecular orbitals involved in the excitation and emission for Pro2 and Pro3 in the absence and presence of Hg^2+^ are investigated in Figure 4 and Figure 5. It shows that the photoabsorption and photoemission processes of the two probes are similar to those of Pro1. Namely, both the excited process and emissive process of Pro2 and Pro3 are mainly localized on the energy donor unit (see Figure 4a and Figure 5a). With the addition of Hg^2+^, there are two mainly allowed transitions for both Pro2+Hg^2+^ and Pro3+Hg^2+^. Moreover, the two transitions are assigned as the energy donor and energy acceptor localized transitions, respectively. Thus, Pro2+Hg^2+^ and Pro3+Hg^2+^ can be stimulated by either exciting the energy donor moiety or the energy acceptor moiety. However, radiative transitions of these molecules are localized on the energy acceptor unit according to the calculations. The above results demonstrate that emission of the energy acceptor moiety of Pro2+Hg^2+^ and Pro3+Hg^2+^ could be obtained by exciting the energy donor moiety. This FRET process is intuitively shown in Figure 4b and Figure 5b.

On basis of the above results, it is interesting to make a comparison among the probes on their sensing performances. It is obvious that the fluorescent emission wavelengths of the probes exhibit great redshifts in the presence of Hg^2+^. In particular, the variation of the energy gap between the two orbitals involved in the emission process for Pro1 and Pro1+Hg^2+^ (0.44 eV) is the largest compared with that of Pro2 and Pro2+Hg^2+^ (0.20 eV) as well as Pro3 and Pro3+Hg^2+^ (0.20 eV), leading the redshift of Pro1+Hg^2+^ versus Pro1 to be the maximum. In addition, by comparing the absorption and emission wavelength, we ascertain the Stokes shifts for the probes and their reaction products with Hg^2+^. All of the molecules present large Stokes shifts that range from 44 nm to 122 nm, with Pro3+Hg^2+^ showing the largest one. An obvious Stokes shift means the avoidance of interference between the absorption and emission spectra. Thus, this is crucial to improving the efficiency of detection. Last but not least, after reacting with Hg^2+^, the fluorescent intensity of Pro1 displays a three-fold enhancement, while the fluorescence enhancements of Pro2 and Pro3 are 2.4-fold and 1.6-fold, respectively, which indicates the preferable sensitivity of Pro1 towards Hg^2+^.

To summarize, the probes Pro1, Pro2 and Pro3 exhibit clear Hg^2+^-induced changes in the excitation and emission features. In addition, the FRET process in Pro*n*+Hg^2+^ (*n* = 1, 2, 3) is demonstrated from the theoretical point of view, suggesting the probes to be promising FRET-based ratiometric and colorimetric fluorescent chemosensors. In particular, Pro1+Hg^2+^ shows the largest redshift in the fluorescent spectrum compared with the fluorescent spectrum of Pro1, and the strongest fluorescence enhancement with obvious Stokes shift relative to Pro2+Hg^2+^ and Pro2+Hg^2+^. As a result, one can anticipate Pro1 to be the superior fluorescent probe in comparison with Pro2 and Pro3.

## 4. Conclusions

The density functional theory in combination with the polarizable continuum model is used to investigate the one- and two-photon absorptions as well as the emission properties of a series of FRET-based fluorescent probes in the absence and presence of Hg^2+^. The effect of the donor on the sensing performance for the studied fluorescent probes is discussed with emphasis. Both the absorption intensity and the fluorescent intensity are enhanced, and the fluorescent wavelengths show visible changes when the probes react with Hg^2+^. Importantly, the TPA cross sections of the compounds are increased with the presence of Hg^2+^, which theoretically suggests the possibility of the probes to be two-photon fluorescent sensors for Hg^2+^. Moreover, the calculated results show that Pro1 exhibits the most remarkable changes on fluorescence with the largest TPA cross section in comparison with Pro2 and Pro3, and the variation of TPA cross sections between Pro1 and Pro1+Hg^2+^ is more obvious. Thus, the energy donor is demonstrated to play a dominant role in the sensing performances on the probes. In addition, Pro1 with naphthalimide derivative as the energy donor is demonstrated to be a preferable chemosensor for Hg^2+^. In addition, the analysis of molecular orbitals is presented to explore the responsive mechanism of the probes, where the FRET processes are intuitively shown in the compounds when they react with Hg^2+^. Our results agree with the available experimental measurements and make a comparison among the probes at the same theoretical level. The present research would be helpful for understanding the relationships of structure with optical properties for the studied fluorescent probes, and would further provide guidelines for designing more efficient fluorescent probes.

## Figures and Tables

**Table 1:** One-photon absorption properties, including the excitation energy *E_opa_* (eV), the corresponding wavelength λ_*opa*_ (nm), the oscillator strength δ_*opa*_ (a.u.), and the transition nature for Pro1, Pro1+Hg^2+^, Pro2, Pro2+Hg^2+^, Pro3 and Pro3+Hg^2+^ in H_2_O. H (L) denotes the HOMO (LUMO).

Molecule	*E_opa_* (eV)	λ_*opa*_ (nm)	δ_*opa*_ (a.u.)	Transition Nature
Pro1	2.85	434	0.35	H-2 → L 98%
Pro1+Hg^2+^	2.53, 2.85	489, 434	0.88, 0.37	H-1 → L 77%, H → L+1 98%
Pro2	2.80	442	0.56	H-2 → L 98%
Pro2+Hg^2+^	2.52, 2.81	491, 440	0.96, 0.58	H-2 → L 98%, H → L+1 98%
Pro3	2.83	436	0.39	H-2 → L 85%
Pro3+Hg^2+^	2.50, 2.90	495, 426	0.73, 0.35	H-1 → L 84%, H → L+1 68%

**Table 2:** TPA properties, including the excitation energy *E_tpa_* (eV), the corresponding two-photon wavelength λ_*tpa*_ (nm), and the TPA cross section σ_*tpa*_ (1 GM = 10^−50^ cm^4^·s/photon) of the lowest five excited states for Pro1, Pro1+Hg^2+^, Pro2, Pro2+Hg^2+^, Pro3 and Pro3+Hg^2+^ in H_2_O.

Molecule	*E_tpa_* (eV)	λ_*tpa*_ (nm)	σ_*tpa*_ (GM)	Molecule	*E_tpa_* (eV)	λ_*tpa*_ (nm)	σ_*tpa*_ (GM)
Pro1	2.71	912	0.00	Pro1+Hg^2+^	2.37	1043	9.18
	2.85	868	534.72		2.38	1039	26.68
	2.87	861	2.83		2.53	977	231.27
	3.13	790	17.81		2.85	868	566.42
	3.36	736	6.15		3.08	802	2502.89
Pro2	2.52	981	2.15	Pro2+Hg^2+^	2.20	1124	6.35
	2.67	926	0.04		2.40	1030	0.00
	2.75	899	108.08		2.52	981	225.73
	2.80	883	33.21		2.65	933	39.48
	3.08	802	0.93		2.81	880	33.51
Pro3	2.51	985	12.10	Pro3+Hg^2+^	2.33	1061	0.00
	2.68	922	10.21		2.50	989	142.91
	2.83	873	89.22		2.58	958	98.54
	2.88	858	15.81		2.85	867	89.89
	2.99	827	49.43		2.90	852	3.09

**Table 3:** Fluorescent emission properties, including the emission energy *E_ope_* (eV), the corresponding emission wavelength λ_*ope*_ (nm), and the oscillator strength δ_*ope*_ (a.u.) for Pro1, Pro1+Hg^2+^, Pro2, Pro2+Hg^2+^, Pro3 and Pro3+Hg^2+^ in H_2_O. H (L) denotes the HOMO (LUMO).

Molecule	*E_ope_* (eV)	λ_*ope*_ (nm)	δ_*ope*_ (a.u.)	Transition Nature
Pro1	2.59	478	0.37	L → H-2 98%
Pro1+Hg^2+^	2.31	535	1.14	L → H-2 98%
Pro2	2.53	489	0.52	L → H-3 87%
Pro2+Hg^2+^	2.30	538	1.23	L → H-2 98%
Pro3	2.46	504	0.64	L → H-2 98%
Pro3+Hg^2+^	2.26	548	1.01	L → H-1 98%
